# L4/5 accessibility for extreme lateral interbody fusion (XLIF): a radiological study

**DOI:** 10.1186/s13018-022-03320-0

**Published:** 2022-11-11

**Authors:** Valentin Quack, Jörg Eschweiler, Christina Prechtel, Filippo Migliorini, Marcel Betsch, Nicola Maffulli, Natalia Gutteck, Markus Tingart, Philipp Kobbe, Miguel Pishnamaz, Frank Hildebrand, Dariusch Arbab

**Affiliations:** 1 Department of Orthopaedics, Trauma and Reconstructive Surgery, RWTH Aachen University Hospital, Pauwelsstraße 30, 52074 Aachen, Germany; 2 Ortho-Centrum Aachen (OCA), Aachen, Germany; 3 Department of Orthopaedic Surgery, Klinikum Mannheim, Mannheim, Germany; 4 Department of Medicine, Surgery and Dentistry, University of Salerno, 84081 Baronissi, SA Italy; 5 School of Pharmacy and Bioengineering, Faculty of Medicine, Keele University, ST4 7QB Stoke on Trent, England; 6 Centre for Sports and Exercise Medicine, Barts and the London School of Medicine and Dentistry, Mile End Hospital, Queen Mary University of London, E1 4DG London, England; 7 Department of Orthopaedics and Traumatology, Martin Luther University Halle Wittenberg, Halle, Germany; 8 Department of Orthopaedic and Trauma Surgery, Klinikum Dortmund, Dortmund, Germany

**Keywords:** Extreme lateral transpsoas approach, XLIF, Lumbar Fusion, Save zone, L4/5

## Abstract

**Introduction:**

Potential advantages of the Extreme Lateral Interbody Fusion (XLIF) approach are smaller incisions, preserving anterior and posterior longitudinal ligaments, lower blood loss, shorter operative time, avoiding vascular and visceral complications, and shorter length of stay. We hypothesize that not every patient can be safely treated at the L4/5 level using the XLIF approach. The objective of this study was to radiographically (CT-scan) evaluate the accessibility of the L4/5 level using a lateral approach, considering defined safe working zones and taking into account the anatomy of the superior iliac crest.

**Methods:**

Hundred CT examinations of 34 female and 66 male patients were retrospectively evaluated. Disc height, lower vertebral endplate (sagittal and transversal), and psoas muscle diameter were quantified. Accessibility to intervertebral space L4/5 was investigated by simulating instrumentation in the transverse and sagittal planes using defined safe zones.

**Results:**

The endplate L5 in the frontal plane considering defined safe zones in the sagittal and transverse plane (Zone IV) could be reached in 85 patients from the right and in 83 from the left side. Through psoas split, the safe zone could be reached through psoas zone II in 82 patients from the right and 91 patients from the left side. Access through psoas zone III could be performed in 28 patients from the right and 32 patients from the left side. Safe access and sufficient instrumentation of L4/5 through an extreme lateral approach could be performed in 76 patients of patients from the right and 70 patients from the left side.

**Conclusion:**

XLIF is not possible and safe in every patient at the L4/5 level. The angle of access for instrumentation, access of the intervertebral disc space, and accessibility of the safe zone should be taken into account. Preoperative imaging planning is important to identify patients who are not suitable for this procedure.

## Introduction

Extreme lateral interbody fusion (XLIF) as a transpsoas approach was introduced by Ozgur and Pimenta as a new less-invasive technique to reduce the complications of existing approaches [1]. Vascular injuries were seen with anterior lumbar interbody fusions (ALIF), muscular and soft tissue trauma with transforaminal lumbar interbody fusions (TLIF), and posterior interbody fusions (PLIF).

XLIF is a minimally invasive procedure performed through the side of the body to treat spinal disorders and reduce long-term back or leg pain that has not responded to other treatments. Potential advantages of the XLIF approach are smaller incisions, preserving the anterior and posterior longitudinal ligaments, lower blood loss, shorter operative time, and avoiding vascular and visceral complications seen with ALIF, TLIF, and PLIF approaches. Furthermore, increased disc space height, shorter length of stay, less pain, lower revision rates, and reduced infection rates have been reported [2–5]. Despite the above-mentioned advantages, neurological and non-neurological complications have been observed [5]. Epstein et al. summarized 13% lumbar plexus injuries, 62.5% irreversible sensory deficits, 0.7–33.6% new motor deficits, and 45% risk of cage-overhang using the XLIF approach [6–8]. Placement of the implant is undertaken without direct visualization of nerve roots and vessels. Structures in danger during discectomy, vertebral endplate preparation, psoas retraction/penetration, and implant insertion are the lumbar plexus, the ventral nerve roots, and retroperitoneal vessels (Guerin I). Several cadaver studies tried to define a safe zone for the XLIF approach to reduce the risk of neural injury. The L4/5 segment in particular is difficult to treat with the XLIF approach because of the local anatomy [5]. Guerin et al. defined a safe working zone to assess neural and vascular anatomic structures in the surgical field using plain radiography and MRI information. They also highlighted that the XLIF approach might be particularly risky at level L4/5 given the closed proximity between the lumbar plexus and intervertebral disc [9, 10].

At the L4/5 level, a more anterior position of the nerve root and a more posterior position of the peritoneal vessels cause a reduction in the safe working zone. Additionally, a high superior edge of the iliac crest limits the potential exposure site to L4/5 which might cause hardware malpositioning, and nerves inside and around the psoas muscle reduce safe transpsoas accessibility. Therefore, accurate knowledge of anatomic relations and appropriate preoperative planning are mandatory before performing the XLIF approach at the L4/5 level.

Not every patient can be safely treated at the L4/5 level using the XLIF approach. This study radiographically evaluated the accessibility of the L4/5 disc space level using an extreme lateral approach considering defined safe working zones and taking into account the anatomy of the superior iliac crest.

## Material and methods

The present study was approved and registered by the ethics committee of the RWTH University of Aachen (project ID EK 015–18) and conducted according to the principles expressed in the Declaration of Helsinki. All patients were able to understand the nature of their treatment and provided written consent to use their clinical and imaging data for research purposes. Patients who underwent computed tomography (CT) examinations between January 2011 and January 2013 were selected from our database. Patients were included if they satisfy t

- Scoliosis < 10°
- Spondylolisthesis < Grade II according to Meyerding
- No previous operations on the lumbar spine section to be operated on
- No significant coxarthrosis:
  - No osteophyte formation
  - No joint space narrowing
  - No irregularities in the articular surface in the CT scan
- No hip arthroplasty (Kepler et al. 2011)
-  > 18 years

he following parameters:

Patients with scoliosis > 10°, spondylolisthesis, history of spine surgery or fracture, hip arthritis or replacement, lumbar infection or tumour, and age < 18 years were excluded. 100 CT examinations of 34 female and 66 male patients were retrospectively evaluated. The mean age was 57.1 (25–93) years. The included patients had undergone the CT examinations for diagnosis of spine pathologies, e.g. spondylolysis. Furthermore, the datasets were used for preoperative planning of the intervention to consider patient-specific parameters.

**Fig. 1:**
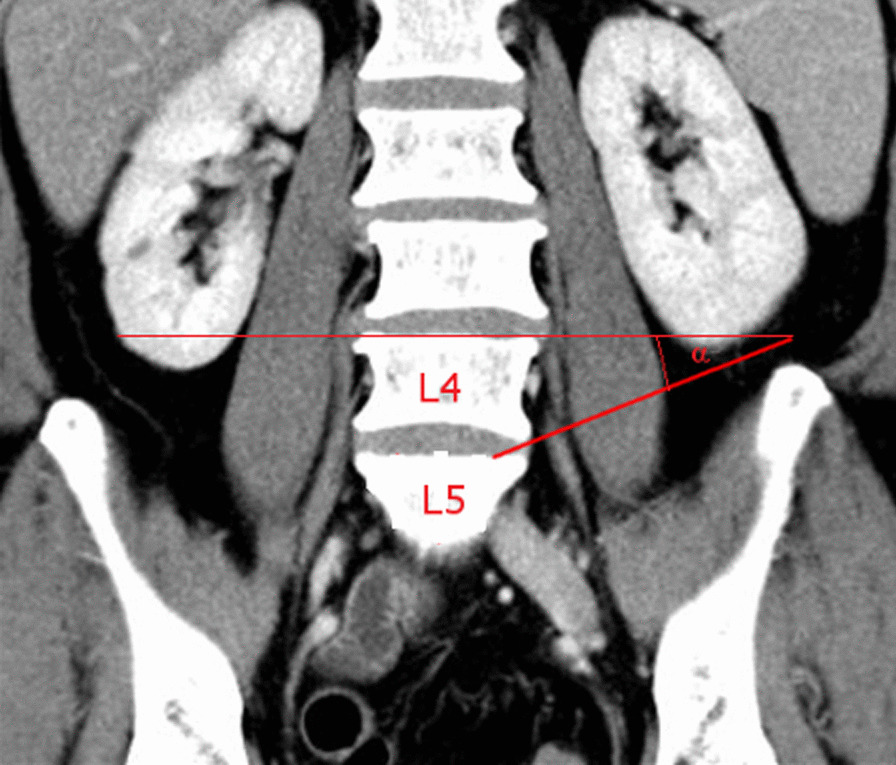
Instrumentation angle α

Two observers (DA and CP) independently performed all measurements twice. Measurements were taken at the L4/5 level using Philips iSite PACS (Version 3.6). Disc height, lower vertebral endplate (sagittal and transversal), and psoas muscle diameter were quantified. Accessibility to the L4/5 intervertebral space was simulated by drawing a line 1 cm cranial from the superior iliac crest to the L5 endplate in frontal cuts. 1 cm relates to instrument size, the L5 endplate was divided into zones I–IV. The angle between lines of the endplate of L4 and L5 was defined as instrumentation angle α (Fig. 1).

**Fig. 2:**
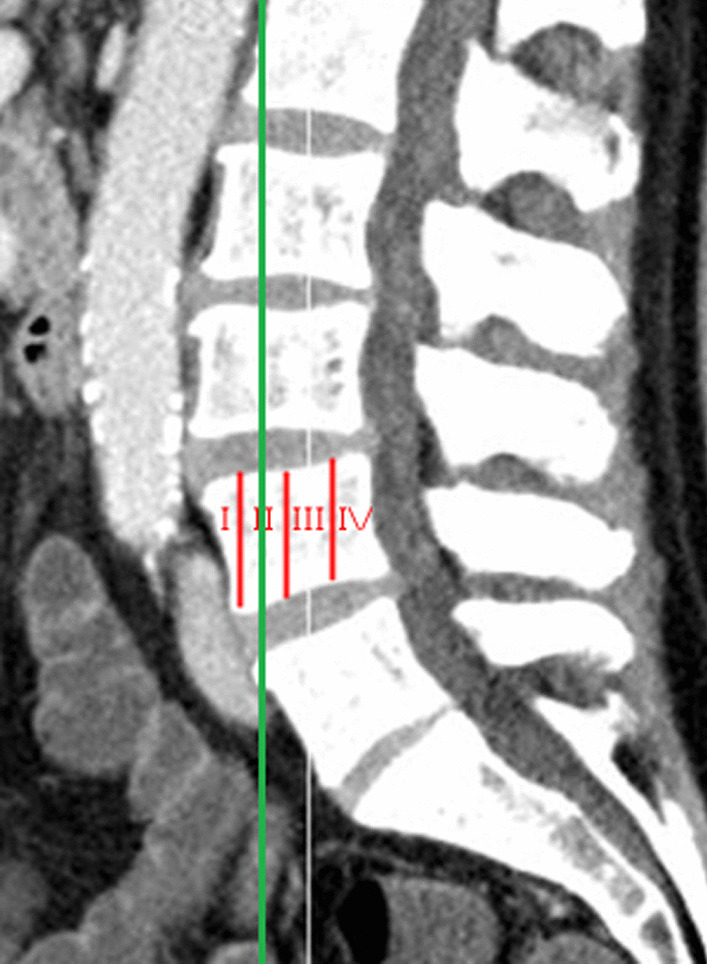
In sagittal scans, L5 was divided into zones I–IV, and zone II was defined as safe

Measurements were obtained from both sides of the spine. A simulated instrumentation line was drawn within the safe zone according to Moro et al. and Guerin et al. [9–11]. The safe zone was determined in sagittal and transverse scans. In sagittal scans, L5 was divided into zones I-IV, and zone II was defined as safe [10] (Fig. 2).

**Fig. 3:**
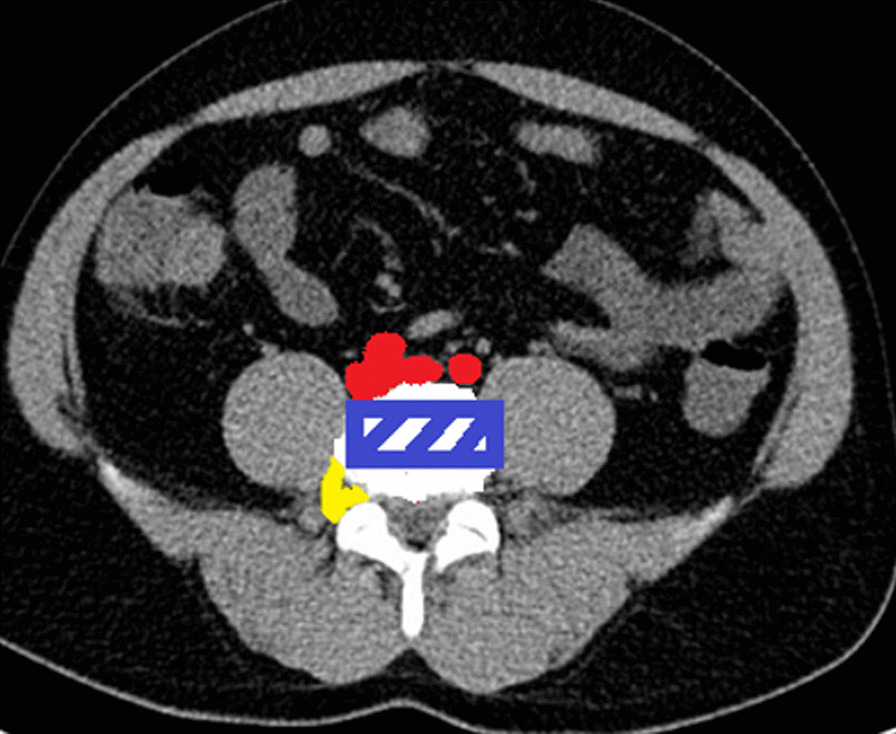
"Safe Zone" in transverse section (rectangle) considering posterior nerve root and anterior vascular positioning according to Guerin et al. (Guerin I)

In transverse cuts, the safe zone was determined as shown in Fig. 3 respecting posterior nerve root position and anterior vascular position [10] from both sides of the spine.

**Fig. 4:**
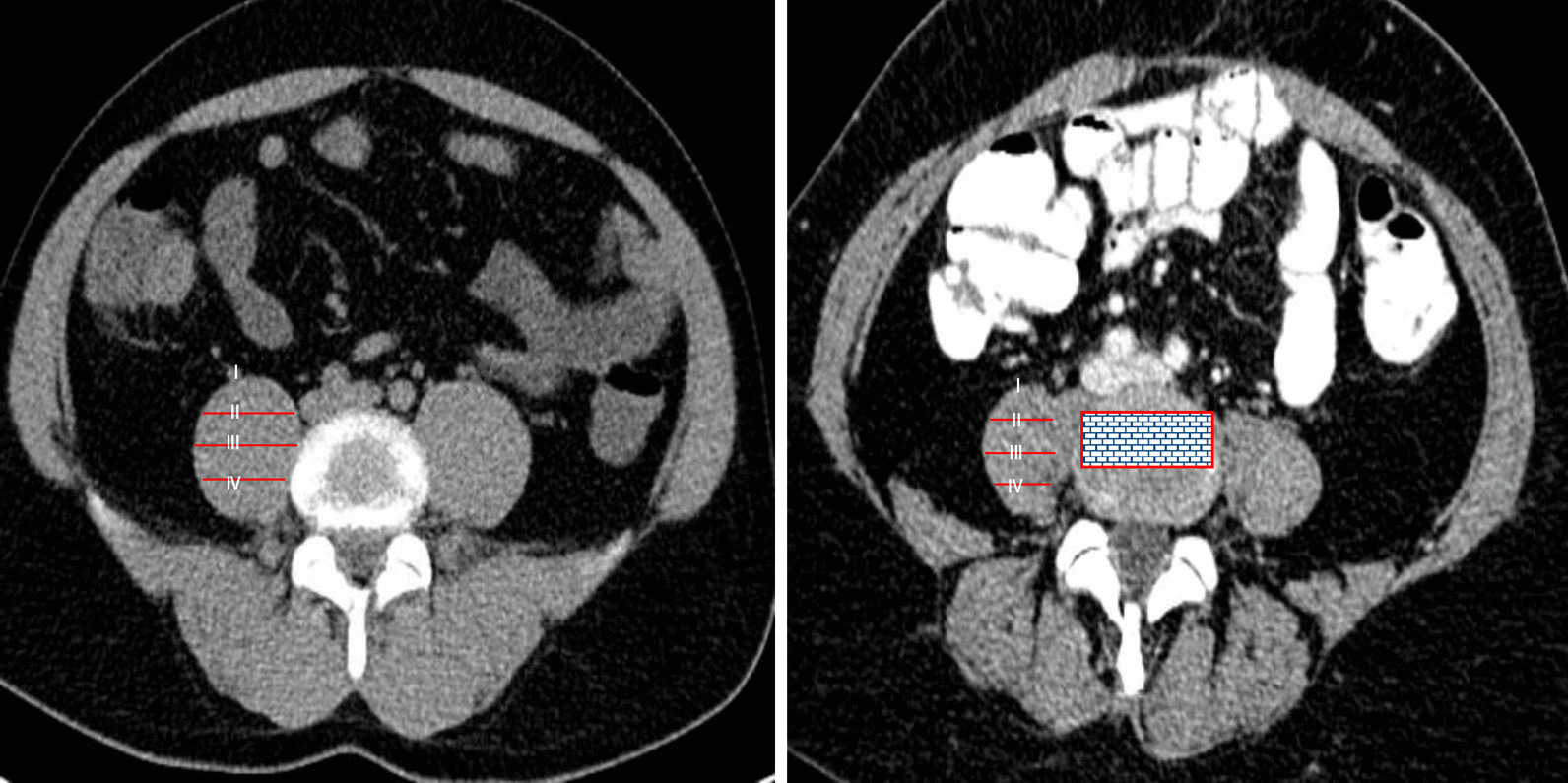
Left: Sectioning of the psoas muscle in 4 zones, where Zone II is defined as safe according to Uribe et al. Right: Final “Safe Zone” in transverse section considering transpsoas access through Zone II and posterior nerve root and anterior vascular positioning

On transverse scans, the psoas muscle was divided into zones I to IV. According to Uribe et al., transpsoas access through zone II and III was defined as safe [12] (Fig. 4). Taking into account the safe zone of sagittal and transverse planes an instrumentation line was drawn into the frontal plane to simulate accessibility.

Safe accessibility and sufficient instrumentation of the lateral approach were defined if the following requirements were met:

- Instrumentation angle in the frontal plane was less than 15°: Complete discectomy is necessary for correct implant positioning and is possible up to 15° of instrumentation angle [12, 13].
- Simulated instrumentation reached zone IV of endplate L5 in the frontal plane representing full access for discectomy
- Simulated instrumentation to the safe zone in the sagittal and transverse planes reached through zone II or III of the psoas.

Statistical analysis was performed using SPSS software (Version 20.0 Armonk, NY IBM Corp). The mean and standard deviation of the magnitudes of the access angles, disc heights, disc widths, lengths and widths of the fifth vertebral body, and diameter of the psoas muscle were determined.

## Results

Disc parameters (vertebral endplate sagittal and transversal diameter, intervertebral height) and psoas diameter are presented in Table 1.

**Table 1:** Overview of the anatomical measurements L5, intervertebral disc, and musculus psoas

(mm)	L5: axial wide	L5: axial depth	Intervertebral disc height L4/5	M. psoas diameter left	M. psoas diameter right
Minimum	36.2	29.3	5.1	30.2	23.9
Maximum	69.7	67.8	16.7	62.6	60
Mean value	56.7	38.7	11.1	44.8	43.5
SD	5.5	5.6	2.2	7.7	8.2

The values of instrumentation angle α are shown in Table 2. An instrumentation angle ≥ 0° means that the intervertebral space L4/5 is at the same level or caudal as the superior iliac crest.

**Table 2:** Distribution of the instrumentation angle α

(Angle in degrees)	Access from the right side	Access from the left side
	Number of patients	Number of patients
α = 0	67	60
α > 0	33	40
α < 15	30	37
α > 15	3	3
Mean value (degrees) of all measured angles α	3	3.3
SD (degrees) of all measured angles α	4.7	4.9

The accessibility of endplate L5 in the frontal plane considering defined safe zones in the sagittal and transverse plane is presented in Table 3. Measurements were obtained from both sides of the spine. Zone IV could be reached in 85% of patients from the right and in 83% from the left side.

**Table 3:** Accessibility of zones I–IV in the coronal section

Zone	I	II	III	IV
Access from the right side	100%	98%	90%	85%
Access from the left side	100%	97%	90%	83%

The accessibility of the safe zone through the psoas split is shown in Table 4. The safe zone could be reached through psoas zone II in 82% of patients from the right and 91% from the left side. Access through psoas zone III could be performed in 28% of patients from the right and 32% from the left side.

**Table 4:** Location of the psoas-splitting (axial section), respecting the safe zone

Zone	I	II	III	IV
Access from the right side	11%	82%	28%	3%
Access from the left side	10%	91%	32%	1%

Safe accessibility and sufficient instrumentation of L4/5 through a lateral approach are shown in Table 5 taking into account all four requirements. 76% of patients could be instrumented from the right, 70% from the left side.

**Table 5:** Accessibility L4/5 considering all requirements

Conditions		Access from the right side (%)	Access from the left side (%)
1	α < 15	97	97
2	Zone IV (Coronal section)	85	83
3	Zone II (Axial section)	82	91
4	Zone III (Axial section)	28	32
1 + 2 + 3 and/ or 4		76	70

## Discussion

Precise knowledge of the anatomic relationship between the lumbar plexus and intervertebral disc is a prerequisite for the safe performance of interbody fusion through a minimally invasive retroperitoneal transpsoas approach. Venous anatomic variants and teardrop-shaped psoas with an anteriorly located plexus may preclude safe access to L4/5 [14]. However, the anatomical location of the L4/5 disc makes access to the disc space difficult and technically challenging [5]. Since the disc space between L4/5 is below the iliac crest in some patients, in these cases angled instruments have to be used.

We investigated the feasibility of minimally invasive XLIF surgery at the L4/L5 level considering the iliac crest, retroperitoneal vessels, and lumbosacral plexus nerves using coronal, sagittal, and axial CT images.

We confirmed our hypothesis that the L4/5 segment is a particular challenge for surgeons through a lateral transpsoas approach. In our study, considering the safe zone described, the access angle, and the adequate reaching of the opposite side, XLIF was only feasible in 76% of patients from the right side and 70% from the left side.

The XLIF technique has been introduced to restore and maintain disc height, restore lordosis, and enlarge the neuroforamen [15–17]. Using XLIF results in indirect decompression of the neural structures and improved intervertebral stability can be achieved through ligamentotaxis [2, 3, 18], avoiding the great vessels and bowel [17, 19, 20].

Given its minimally invasive, XLIF further reduces access morbidity, postoperative pain, and hospital stay and allows rapid return to daily living activities [21–23]. Furthermore, excellent fusion rates have been described [19, 20].

However, a remarkable increase in neurological complications was recorded using XLIF compared to anterior or posterior fusion procedures. Plexus injuries (13–28%), sensory deficits (0–75%), motor deficits (0.7–33.6%), anterior thigh pain (12.5–25%), and sympathectomy (4–8%) have been described [7, 8, 6, 24]. Because of this, anatomical studies have attempted to define safe working zones for the XLIF approach, thereby reducing the risk of neurological complications.

Guerin et al. defined a safe zone ventral to the nerves and dorsal to the great vessels. They were able to show that the area of this zone decreases steadily from L1/2 to L4/5 and is about half as large in L4/5 compared to L1/2 [10, 25].

In an anatomical cadaver study, they further demonstrated that the safe zone shifts ventrally from L1/2 to L4/5. For this purpose, they divided the disc into four zones (1: anterior quarter; 2: middle anterior quarter; 3: middle posterior quarter; 4: posterior quarter). A safe working zone was defined by the absence of crossing off a lumbar plexus branch. The safe working zone includes zones 2 and 3 for L1/2, zone 3 for level L2/3, zone 3 for level L3/4, and zone 2 for level L4/5. They concluded that the transpsoas approach L4/5 is particularly challenging and risky given the anatomical relationships between the lumbar plexus and disc. Alternative approaches such as TLIF, PLIF, or ALIF should be used [10, 25].

Uribe et al. define the safe working zone in terms of the lumbar plexus branches. They investigated this in five cadaveric specimens. In their study, the safe zones at the disc from L1/2 to L3/4 were in the middle of zone 3, and the safe zone at L4/L5 was at the boundary between zone 2 and 3 [12].

Some authors pointed out that there is no absolute safe zone, and they would recommend either direct visualization of the nerve and/or the use of neuromonitoring [26]. Also, the L4/5 level presents an increased risk of intraoperative nerve and vessel injury because the nerve roots of the lumbosacral plexus run more anteriorly than in the cranially located intervertebral disc spaces and the retroperitoneal vessels run directly in front of the vertebra [27].

Furthermore, the anatomical location of the L4/5 disc space to the iliac crest presents a challenge to the surgeon. Based on direct operative experience in XLIF access surgery, Pimenta et al. defined a maximum access angle of 15° for a lateral retroperitoneal transpsoas approach at the L4/L5 level. This angle can be accommodated by angled instruments, which can be used when removing the disc at the L4/5 level [28]. In the present study, an access angle α smaller than 15° was measured in the CT images from both the right and left sides in 97 patients. Among the 97 patients, an angle α equal to 0° was measured in 67 patients when accessed from the right side and in only 60 patients when accessed from the left side. In contrast, an angle α less than 15° was determined in 30 patients on the right and 37 patients on the left. This difference between an approach from the right and left side possibly arises from the fact that the patients did not lie in the computed tomography scanner with an upright spine.

In 2013, Fontes et al. studied the lateral retroperitoneal transpsoas approach at the L4/5 level whishing to ascertain how often the iliac crest prevents an XLIF using 20 cadavers [29]. In 13 of 20 candidates (65%), the disc space at the L4/5 level could be completely instrumented through a lateral transpsoas approach [29]. In the present imaging study, accessibility of the disc space with angled instruments could be demonstrated in 97% of patients. However, Fontes et al. did not use angled instruments [29]. Therefore, the disc level L4/5 had to align perfectly with the instruments. In our study, this would be the case in patients with an angle α equal to 0°. Thus, the results are comparable [1].

The transpsoas access is not safe at every location. In our study, based on its diameter, the psoas muscle was divided into four equal zones (Fig. 4a). The genitofemoral nerve is located in the anterior part (zone I), and other nerves of the lumbosacral plexus (iliohypogastric nerve, ilioinguinal nerve) are located in the dorsal part (Zone IV) of the psoas [11]. Therefore, splitting of the muscle in zone I or IV may result in injury to the nerves. Furthermore, the retroperitoneal vessels are also located in zone I and there is a high risk of injury to the vessels splitting the psoas in zone I [10, 25, 12]. The blue-shaded box in Fig. 4b represents the "Safe Zone".

We also evaluated how the “safe zone” of the psoas muscle can be reached in axial sections.

Taking into account all three conditions, our study suggests that the XLIF is only feasible in 76% of patients from the right and 70% from the left side.

Therefore, precise preoperative planning is essential to avoid complications in XLIF. However, intraoperative neuromonitoring is mandatory to avoid neurological complications [17, 26]. In patients in whom the L4/5 space cannot be safely reached using XLIF based on preoperative planning, another lumbar interbody fusion (LIF) option should be used. In a cadaveric study, Fontes et al. performed an iliac crest osteotomy when needed and were able to complete all XLIFs (13/20 without and 7/20 with osteotomy) [29]. Whether this should be routinely performed is questionable. Oblique lumbar interbody fusion (OLIF) is a possible alternative for the ventral operative treatment of the L4/5 level.

To eliminate negative influencing factors on the radiographic measurements as far as possible, only the appropriate section was included. This positively changed the type of patient that fulfilled the defined imaging prerequisites for performing an XLIF in this study. Patients who were not selected for the study because of the exclusion criteria could negatively influence the results of the study. The measurements of the present study were taken on CT scans. The lack of a control group is another limitation of the study.

The accessibility of the intervertebral disc depends on the anatomy of the iliac crest and the anatomy of the psoas major muscle. The influence of criteria such as the height of the disc, the width of the cover plate, and the diameter of the psoas major muscle were not analysed in greater detail.

The CT scans of the patients were not performed in an extreme lateral position. Accuracy in measuring access angles could be increased if the patient's CT scans were performed in an extreme lateral position.

The direct transfer of the acquired information to clinical use is not fully possible. The position in which the CT scans were performed is different from positions in activities of daily living and real-life situations, respectively. The information could nevertheless be used for preoperative planning of the intervention because the surgical reconstruction often takes place in a similar position and orientation as the imaging. Furthermore, the images give a good overview of the pathologies and the current (anatomical) variations so that the surgeon could include this in his (surgical) decisions.

Furthermore, the demographic data of the patients should be considered. Equal numbers of men and women were not selected and 34 women and 66 men were included in the study. The male iliac crest is anatomically different from the female iliac crest. We do not know to what extent our results would have changed if the genders had been equally represented.

## Conclusion

XLIF is a minimally invasive approach, which, however, is not suited to every patient at the L4/5 level. Precise preoperative planning is extremely important. The angle of access for instrumentation, accessibility of the contralateral portion of the intervertebral disc space, and accessibility of the safe zone should be taken into account. In cases where XLIF cannot be safely performed because of anatomical conditions, OLIF or other LIF techniques should be considered.

## Data Availability

The datasets analysed during the current study are not publicly available due to limitations of ethical approval involving the patient data and anonymity but are available on reasonable request. Please contact Valentin Quack (vquack@ukaachen.de) for data request.

## Abbreviations

XLIF: Extreme lateral interbody fusion
ALIF: Anterior lumbar interbody fusions
TLIF: Transforaminal lumbar interbody fusions
PLIF: Posterior interbody fusions
CT: Computed tomography
